# Symptomatic Patients With Inflammatory Bowel Disease Do Not Always Require Treatment With Corticosteroids

**DOI:** 10.1016/j.gastha.2026.100911

**Published:** 2026-03-03

**Authors:** Gal Hodish, Jeffrey Berinstein, Shirley Cohen-Mekelburg

**Affiliations:** 1 Division of Gastroenterology & Hepatology, Michigan Medicine, Ann Arbor, Michigan; 2 VA Center for Clinical Management Research, U.S. Department of Veterans Affairs, Ann Arbor, Michigan

Up to 30% of patients with inflammatory bowel disease (IBD) experience flares each year requiring treatment with corticosteroids or advanced therapies.^1^, ^2^, ^3^, ^4^ However, diarrhea and abdominal pain, which are hallmarks of IBD, may instead be driven by noninflammatory conditions mimicking IBD. In fact, gastrointestinal symptoms correlate poorly with objective inflammation in IBD.^5^^,^^6^ Therefore, clinicians must maintain a broad differential diagnosis and distinguish inflammatory from noninflammatory symptoms before escalating IBD therapy.^7^

The differential diagnosis for an IBD patient with abdominal pain and diarrhea includes active inflammation, *Clostridiodes difficile* and other enteric infections, microscopic colitis, bile acid diarrhea, fibrotic strictures, absorptive disorders (eg, celiac disease), and disorders of gut-brain interaction.^8^^,^^9^ For example, in 1 study, up to 17% of symptomatic patients with IBD tested positive for a non–*C difficile* gastrointestinal infection.^8^ Furthermore, patients with IBD and a negative workup may benefit from disorders of gut-brain interaction-directed antidiarrheal treatment.^9^ Finally, patients with a fibrotic stricture may require dietary modification or surgery.

In conclusion, it is important to maintain a broad differential diagnosis for symptomatic patients with IBD. Objective testing is often necessary to guide appropriate treatment targeting the underlying inflammatory or noninflammatory condition.
